# Characterization of immune cell populations in syngeneic murine tumor models

**DOI:** 10.1002/cam4.5784

**Published:** 2023-03-13

**Authors:** Sofie Snipstad, Frida Bremnes, Mats Dehli Haugum, Einar Sulheim

**Affiliations:** ^1^ Department of Biotechnology and Nanomedicine SINTEF Industry Trondheim Norway; ^2^ Department of Physics Norwegian University of Science and Technology Trondheim Norway; ^3^ Cancer Clinic St. Olavs Hospital Trondheim Norway; ^4^ Department of Pathology St. Olav's University Hospital Trondheim Norway

**Keywords:** animal models, checkpoint inhibitor, immunotherapy, tumor immune microenvironment

## Abstract

Immunocompetent murine models are important tools for preclinical evaluation of immunotherapies. Here, six different immunocompetent tumor models based on four different cell lines were characterized, including metastatic lung cancer (CMT 167), triple‐negative breast cancer (4T1), pancreatic cancer (KPCY), and colon cancer (MC38). The tumors were implanted subcutaneously or orthotopically before the animals were treated with anti‐PD1 checkpoint inhibitor. A range of innate and adaptive immune cells were then quantified by flow cytometry of single‐cell suspensions from the tumors. Furthermore, confocal laser scanning microscopy was used to quantify the density and distribution of T‐cells in frozen sections. A model‐dependent cellular immune landscape was observed, with variable responsiveness toward anti‐PD1, ranging from the most responsive MC38 colon cancer model to the least responsive 4T1 breast cancer model. The study provides an overview of the immune landscape of these tumor models, and a foundation for further elucidation of pro‐tumor and anti‐tumor mechanisms behind heterogeneous responses towards immunotherapies.

## Graphical abstract

In this study we have evaluated six different murine tumor models for immune cell content and response to checkpoint inhibition. Tumors were characterized using both flow cytometry and microscopy and the models differed substantially in presence of immune cells and in response to anti‐PD1 antibody.

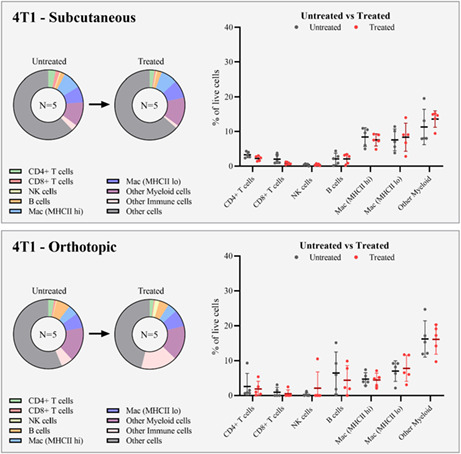

## 1 INTRODUCTION

Progress in cancer research depends heavily on adequate modeling systems to test hypotheses and optimize treatment strategies prior to clinical trials.^1^ While human cancer cells implanted in immune‐compromised mice have been the standard for decades, syngeneic models, where murine cells are implanted in mice, have grown in popularity with the increasing focus on the role of the immune system both in cancer growth and therapy. Syngeneic models in mice are especially useful for mechanistic studies related to drug delivery and immunology. In these studies, the biology and physiology of the tumors can be more important than tissue of origin and similarity to its counterpart in human cancer.

Trials in murine models of cancer benefit greatly from in‐depth knowledge about the complex microenvironment of the tumor models used. We have previously characterized the microenvironment in different human cancer models in mice^2^ and various syngeneic models have been characterized and compared by others.^3^ To facilitate use of and translation from experimentation with syngeneic tumors, we have characterized six tumor models and their response to PD‐1 therapy. The models are based on four different cell lines and were evaluated by histopathology, using flow cytometry to map immune cell content, and by fluorescence imaging to assess T‐cell distribution in the tumor. The tumor models 4T1 (mammary carcinoma, subcutaneous [SC] and orthotopic [OT] implantation), CMT 167 (lung carcinoma, SC implantation and lung metastases [intravenous (IV) injection]), KPCY (pancreatic ductal adenocarcinoma, SC implantation) and MC38 (colon carcinoma, SC implantation) were included. These models are frequently used syngeneic models, but a comparison and a characterization of their immune landscape are lacking from the literature.

The triple negative 4T1 is derived from a spontaneous tumor in mammary gland of a BALB/c mouse. Upon implantation, 4T1 forms tumors that are invasive, poorly immunogenic and spontaneously metastasizing to distant sites such as lymph nodes, lungs, liver, brain and bone.^4^ CMT 167 is a highly metastatic murine alveogenic lung carcinoma derived from CMT 64 cells, which were isolated from a primary lung tumor in a C57BL/lrf mouse.^5^ The cell line metastasizes from the primary tumor but is in this study used as a lung metastases model based on IV injection of the cells. The KPCY cells are derived from autochthonous tumors in C57BL/6 KPC mice. KPC mice are genetically modified to express mutant KRAS and P53 in the pancreas and develop spontaneous pancreatic ductal adenocarcinomas sharing multiple features with human tumors. The cells frequently metastasize to liver and lungs. Cells from these tumors can be isolated and maintained in culture and also separated into submodels based on features such as immunogenicity and metastasizing potential.^6^ In this study, the KPCY clone 2838c3 was used. MC38 is a metastatic cell line isolated from colon adenocarcinoma in a C57BL/6 mouse induced by carcinogen exposure.^7^ The model is often used for testing of combination treatment with immunotherapy as it responds to immunotherapy.

These cell lines and corresponding tumor models are all frequently used in preclinical oncology research and are verified as useful and relatively representative models of human cancer. However, tumor model comparisons are infrequently published and comparison between different studies is challenging due to differences in experimental setup and analyses. In this paper, we aim to give a thorough evaluation of the pathological assessment and immunological landscape of these popular tumor models.

## 2 MATERIALS AND METHODS

### 2.1 Cells and medium

CMT 167 cells (kind gift from Prof. Paola Allavena, Humanitas Research Hospital) were cultured in Gibco Dulbecco's Modified Eagle Medium (DMEM), high glucose (Sigma‐Aldrich, #D5796), with 10% Fetal Bovine Serum (FBS, Sigma‐Aldrich #F7524), 2 mM L‐glutamine and 100 U/mL penicillin–streptomycin (Pen/strep). 4T1 cells (CRL‐2539, American Type Culture Collection (ATCC)) were grown in RPMI 1640 Medium with 10% FBS and 100 U/mL Pen/strep. KPCY cells (2838c3, Kerafast) were cultured in DMEM (Sigma‐Aldrich #D5796) with 10% FBS and 100 U/mL Pen/strep. MC38 cells (ENH204, Kerafast) were cultured in low glucose DMEM (Sigma‐Aldrich #D6046) with 10% FBS, 2 mM L‐ glutamine, 1* non‐essential amino acids, 1 mM sodium pyruvate, 10 mM HEPES (4‐(2‐hydroxyethyl)‐1‐piperazineethanesulfonic acid), and 100 U/mL Pen/strep. All chemicals were from Sigma‐Aldrich unless specified. Cells were thawed and maintained in exponential growth for 2–3 weeks at 37°C and 5% CO_2_ before implantation.

### 2.2 Mice and cell implantation

CMT 167, KPCY, and MC38 models were established in 8‐week‐old female C57BL/6 mice (Janvier Labs), while 4T1 models were established in 8‐week‐old female BALB/c mice (Janvier Labs). Subcutaneous implantation was performed by injecting a set amount of tumor cells suspended in growth medium under the skin on the left hind leg of each mouse, resulting in the four subcutaneous models, CMT 167 SC (100,000 cells in 50 μL per mouse), 4T1 SC (10,000 cells in 50 μL per mouse), KPCY SC (300,000 cells in 120 μL per mouse), and MC38 SC (500,000 cells in 50 μL per mouse). Orthotopic models were also established for the CMT 167 and 4T1 cell lines by implanting the suspension into the organ from which the cell line originates. The CMT 167 OT model was established by intravenous injection in the tail vein (200,000 cells in 100 μL per mouse), resulting in lung metastases. The 4T1 OT model was established by injecting 4T1 cell suspension into the third mammary fat pad of each mouse (10,000 cells in 50 μL per mouse). The mice were anesthetized during implantation by inhalation of 2–3% isofluorane (Baxter). Body temperature was maintained using a heating pad during all procedures. Body weight and clinical symptoms were monitored twice weekly, in addition to tumor size as measured by calipers. The animals were housed in groups of 5 in individually ventilated cages in specific pathogen‐free conditions. The cages were enriched with housing, nesting material, bedding, and gnaw sticks, and were kept at 20–23°C with 50–60% humidity at a 12 h night/day cycle. All animals had free access to food and sterile water. The animal experiments were approved by the Norwegian Food Safety Authorities under FOTS application #24979.

### 2.3 PD‐1 treatment

Half the animals were treated with checkpoint inhibitor PD‐1 which prevents inhibition of T‐cell function. The mice belonging to the treated groups received 10 mg/kg anti‐PD1 antibody (BioXcell, BD0146) twice a week by intraperitoneal injection for a total of four treatments, while the untreated animals received saline injections of equal volume (50 μL per animal). When one or more of the animals had a tumor that reached the predefined endpoint of tumor length of approximately 10–15 mm, all the mice with that tumor type were anesthetized and euthanized. For all tumor types, this occurred 10–18 days after implantation.

### 2.4 Tumor sectioning

**FIGURE 1:**
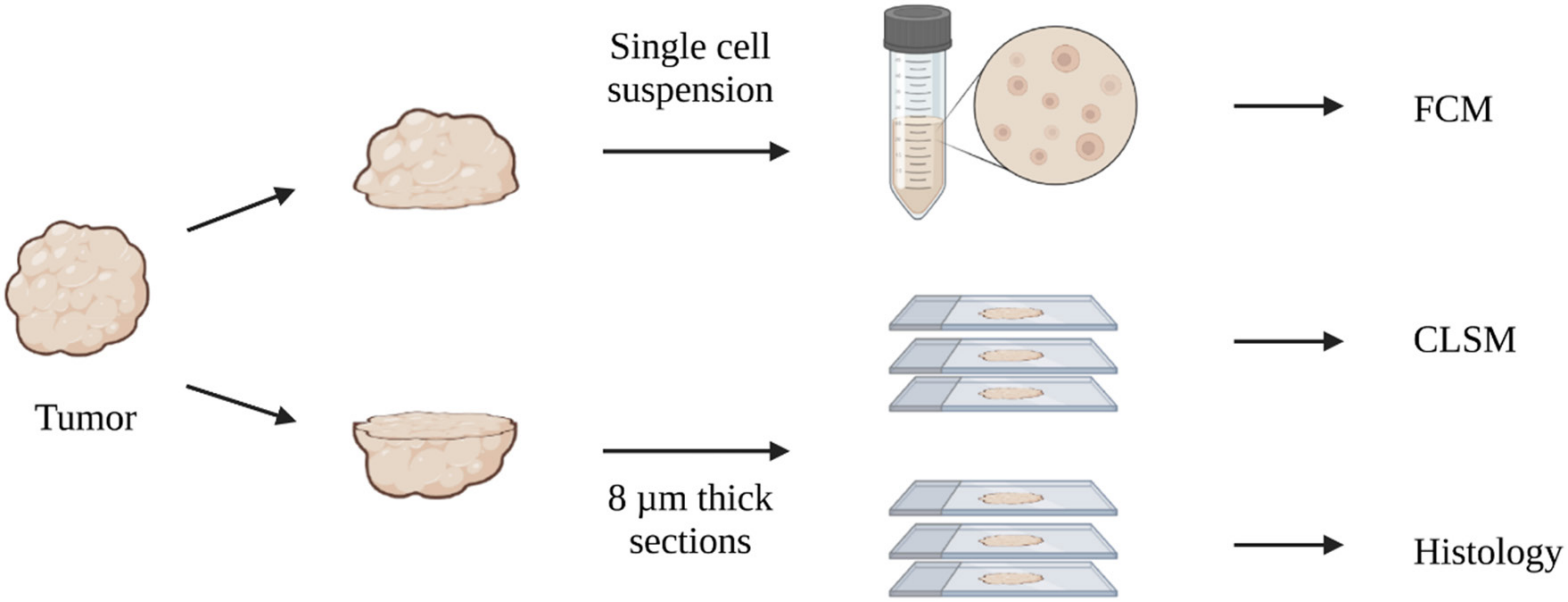
Study overview. Tumor tissue was cut in two, one part for single‐cell suspensions and subsequent analysis by flow cytometry. The other part was frozen and sectioned and imaged by confocal microscopy after staining.

Subcutaneously implanted tumors were surgically removed from the hind leg, separated from the surrounding normal tissue, and divided into two halves along the middle (Figure 1). Half of the tumor was used for single‐cell analysis by flow cytometry (FCM), and the other half was used for immunohistochemistry (IHC) and analysis by confocal laser scanning microscopy (CLSM). The half which was used for FCM was submerged in medium until further processing. The other half was mounted on a piece of cork with OCT Tissue Tek (Sakura) before being submersed in liquid nitrogen following storage at −80°C until sectioning. The orthotopic breast cancer tumors (4T1 OT) were treated in the same way as the subcutaneous tumors, as the primary tumors were possible to separate from the normal tissue of the mammary fat pad. For the orthotopic lung tumors (CMT 167 OT), the left lung was used for sectioning and CLSM analysis and the remaining lobes for FCM. The frozen tissue was sectioned into 8 μm sections, of which two were fixed and stained with hematoxylin, erythrosin, and saffron (HES).

### 2.5 Staining of sections and confocal imaging

The frozen sections were stained using antibodies described in Table 1. Before staining, the sections were fixed in acetone (−20°C) for 10 min, washed in phosphate buffered saline (PBS), and blocked using 12% bovine serum albumin (BSA) in PBS for 60 min at room temperature. After washing in PBS, the sections were incubated sequentially with antibodies at room temperature for 60 min, followed by washing 3× with PBS after staining. Sections were then mounted using Vectashield Vibrance Antifade Mounting Medium with 4′,6‐diamidino‐2‐phenylindole (DAPI, Vector Laboratories).

**TABLE 1:** Antibodies used for staining of frozen sections.

Antibody	Target	Dilution
Primary		
Rabbit anti‐CD3 (Abcam #ab5690)	CD3	1:100
Rat anti‐CD8 (Abcam #ab22378)	CD8	1:250
Secondary		
Alexa Fluor 488 anti‐rabbit (Dianova #711–546‐152)	Rabbit IgG	1:50
Alexa Fluor 647 anti‐rat (Dianova #712–605‐153)	Rat IgG	1:50

Images of the stained sections were acquired with a confocal laser scanning microscope (Zeiss LSM 800), using a Plan‐Apochromat 20×/0.8. All samples were imaged within 12 h after being mounted, and imaged using identical settings optimized to obtain maximum signal with minimum background. AF‐488 was excited at 488, and fluorescence was detected at 515–620 nm. AF‐647 was excited at 640 nm while emission was detected at 656–700 nm. DAPI was excited at 405 nm while detecting fluorescence at 400–515 nm.

### 2.6 Image analysis

Images were analyzed in Image J. Tumors were segmented into two ROIs based on a manually traced outline; Invasive margin (IM), defined as the first 500 μm reaching inwards from the tumor surface, and core tumor (CT) defined as everything inside the IM. The HES‐stained section of the tumor was actively used during the tracing of the tumor outline to distinguish between tumor tissue and normal tissue. For the CMT 167 lung metastases model, tumors were semi‐automatically segmented using color segmentation of HES‐stained sections in ImageJ. The resulting mask was transferred to the frozen section for segmentation of the fluorescence images.

Semi‐automatic thresholding and particle analysis were used to quantify the density of T cells and CD8+ T cells in the two tumor regions as the #cells per mm^2^ tumor. Since CD3 is a pan T cell marker expressed by all T cells, the signal from the CD3 channel was used to create a binary mask that represents the individual T cells, termed the T cell mask. The T cell mask generated based on the CD3 channel was then used to measure the mean gray value of the CD8 channel for each T cell. Visual inspection of several hundred cells across all tumor models was used to determine an appropriate threshold for the mean gray value in the CD8 channel. The cells with a mean gray value above the set threshold were counted as CD8+ T cells. Artifacts such as rips, holes, out‐of‐focus areas, and areas with high background fluorescence were excluded from analysis.

### 2.7 Pathological assessment

HES‐stained sections were evaluated by a clinical pathologist. Imaging of the HES‐stained sections was performed as a tile scan with a Zeiss LSM 800 confocal microscope. The tile scans were imaged in bright‐field with a Plan‐Neofluar 10× objective with a numerical aperture of 0.30.

### 2.8 Single‐cell suspensions and flow Cytometry

To prepare for flow cytometry, tumors were first manually minced to pieces <1 mm and incubated for 65 min at 37°C in 21.5 μL/mL Liberase DL (Roche #5401160001), 21.5 μL/mL Liberase TL (Roche #5401020001), and 13.75 μL/mL DNase 1 (Qiagen #79254) in PBS. PBS with BSA was added to stop disintegration before the suspensions were filtered through a 70‐μm cell strainer (Falcon), centrifuged (390 RCF for 5 min), and resuspended in PBS. Before antibody staining, live cells were counted using trypan blue (Invitrogen #T10282) and Fc receptors were blocked using Mouse Seroblock FcR (Bio‐rad #BUF041A, 3.23 μL/mL) for 15 min. Cells from each tumor were then split into two tubes and stained with one of the two antibody panels from Table 2 for 60 minutes. The cells were then washed twice by centrifugation and resuspension in BSA PBS.

**TABLE 2:** Antibodies for flow cytometry.

Panel	Antibody	Target population	Dilution
A & B	Anti‐CD45 (BD Biosciences, 557235; Clone 30‐F11)	Immune cells	1:100
A & B	Anti‐CD11b (eBio‐ sciences, 47‐0112‐82; Clone M1/70)	Myeloid cells	1:200
A	Anti‐NK1.1 (eBiosciences, 12‐5941‐82; Clone PK136)	NK cells	1:50
A	Anti‐ CD3 (eBiosciences, 17‐0031‐82; Clone 145‐2C11)	T‐cells	1:62.5
A	Anti‐CD4 (BioLegend, 100510; Clone RM4‐5)	CD4+ T cells	1:75
A	Anti‐CD8a (eBiosciences, 17‐0031‐82; Clone 145‐2C11)	CD8+ T cells	1:250
B	Anti‐F4/80 (BioRad, MCA497PE; Clone Cl:A3‐1)	Macrophages	1:25
B	Anti‐CD206 (biolegend, 141707; Clone C068C2)	M2‐like macrophages	1:60
B	Anti‐MHCII (BioLegend, 107616; Clone M5/114.15.2)	MHCII high macrophages	1:250
B	Anti‐CD19 (BioLegend, 115519; Clone 6D5)	B cells	1:122

**FIGURE 2:**
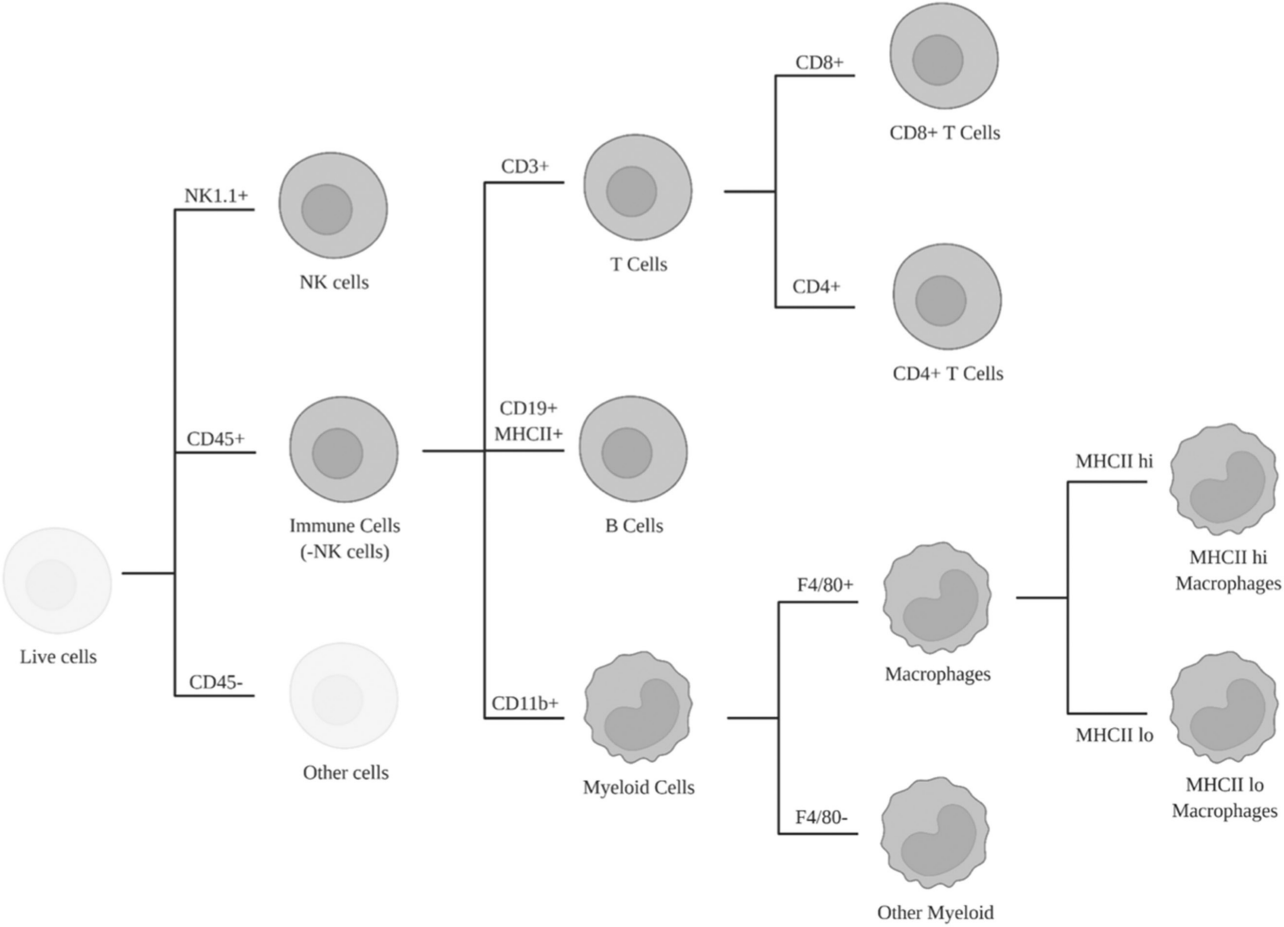
Gating strategy applied after flow cytometry to identify subpopulations of immune cells.

Cells were then stained with a live/dead stain (Fixable Aqua Dead Cell Stain Kit, Thermo Fisher #L34957, 1:1000) for 30 min before being washed and resuspended in cold PBS with 1% BSA for flow cytometry. Flow cytometry was performed on a Gallios Flow Cytometer (Beckman Coulter) using four lasers and detectors for forward and side scatter in addition to seven channels for fluorescence as shown in Table 3. The cells were then gated for singlets and defined into different populations using the gating strategy shown in Figure 2 using FlowJo (BD Life Sciences).

**TABLE 3:** Fluorophores used for FCM together with the respective lasers and detectors used.

Target (Panel A/B)	Dye (Panel A/B)	Excitation	Detector
Dead cells	Aqua	405	550/40 nm
CD45	PerCP (peridin‐chlorophyll‐protein)	488	675/20 nm
CD11b	APC (allophycocyanin)‐eFLuor780	633	775/LP nm
NK1.1/F4/80	PE (phycoerythrin)	561	575/20 nm
CD3/CD206	APC	633	660/20 nm
CD4/MHCII	FITC (fluorescein isothiocyanate)/Alexa Fluor 488	488	525/20 nm
CD8/CD19	PE‐Cyanine7	488 + 521	775/LP nm

All samples from panels A and B were subject to a series of the same gating steps; gating for singlet events, live cells, and all immune cells (CD45+). Most populations were defined by co‐expression of several markers including the expression of CD45 which is expressed on most immune cells (except for NK cells which were gated directly from live cells). T cells were gated from CD45+ cells in panel A as CD3+ and then as either CD4+ or CD8+. B cells were gated from CD45+ cells in panel B by co‐expression of CD19 and MHCII. Myeloid cell populations were based on the gating of all immune cells (CD45+) and myeloid cells (CD11b+) before further gating for macrophages (F4/80+) and other myeloid cells (F4/80−). Macrophages were further divided into two subgroups based on the expression of MHCII (low/high).

### 2.9 Analysis

Data analysis was performed using GraphPad Prism version 9.3.1 for Windows. Two‐way ANOVA was used to perform an inter‐model comparison of untreated, subcutaneous tumors from all four cell lines (CMT 167 SC, 4T1 SC, KPCY SC, and MC38 SC). Tukey's test was used to correct for multiple comparisons, and individual variances were computed for each comparison. Multiple *t*‐tests were used for comparing untreated and treated groups (e.g., 4T1 SC untreated vs. 4T1 SC treated). T‐tests were also used to compare the baseline immune cell populations of orthotopic and subcutaneous tumors originating from the same cell line (e.g., 4T1 SC untreated vs. 4T1 OT untreated). An unpaired t‐test was used, assuming a Gaussian distribution and equal standard deviation in the groups compared. The significance level was set to *p* < 0.05.

## 3 RESULTS

### 3.1 Histological evaluation

**FIGURE 3:**
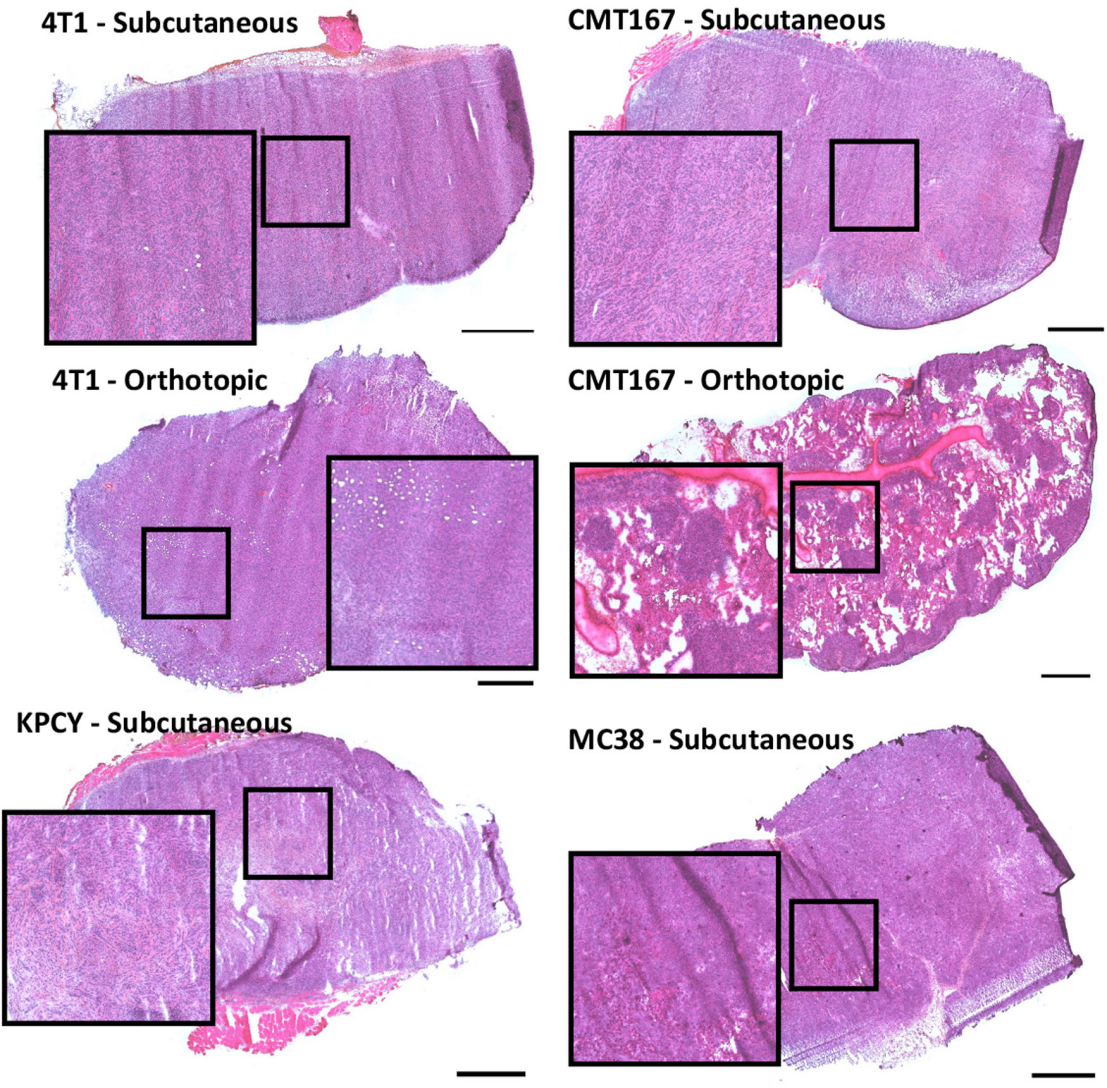
Representative HES‐stained sections from each of the tumor models. Scale bars are 1000 μm.

Sections stained with HES for histopathological evaluation are shown in Figure 3. Histopathologically the tumors are principally very similar, all fitting into the category of poorly differentiated or undifferentiated tumor. Nevertheless, some identifying features could be described for each tumor. 4T1 tumors were implanted both subcutaneously and into the mammary fat pad. The two models showed similar growth patterns, with largely diffuse growth and cells arranged in strings along slender fibrous bands. In both models, fibers of striated muscle and adipose cells were seen incorporated into the tumor. Additionally, in the orthotopic model, focal incorporation of mammary glandular tissue was seen. In the orthotopic model, necroses were larger and more abundant. Inflammation was sparse in both models.

CMT‐167 was grown either as subcutaneous tumors or as lung metastases. In both models, the tumor growth pattern consisted of small clusters, islands and strings of tumor cells. The subcutaneous model showed considerably larger amount and thickness of fibrous bands than the metastasis model. It also showed adipocytes and muscle fibers incorporated into the edge of the tumor. In the metastasis model, tumors were smaller, appeared less cohesive with less fibrous tissue and showed more inflammatory cells within tumors.

KPCY showed a growth pattern similar to CMT‐167, with tumor cells arranged in clusters, islands and strings, as well as glandular structures, interspersed by variably thick bands of fibrosis. KPCY showed the most prominent infiltration of immune cells, with both lymphoid cells and, most prominently, neutrophilic granulocytes dispersed widely throughout the tumors. Muscle fibers and adipose cells were incorporated into the tumors at the periphery.

MC‐38 showed a largely solid or diffuse growth pattern, with the least amount of fibrous tissue. Accordingly, the tumor also showed a much softer texture and providing sections of the tumor was more challenging than for the other models. The model showed relatively sparse inflammation, but a very high density both of cells undergoing mitosis and of apoptotic cells. The tumors showed multiple areas of necrosis. Incorporation of muscle fibers into the tumor was seen focally at the edge of one of the tumors.

The CMT167 and KPCY tumors showed considerably more fibrous tissue than the 4T1 and MC38 tumors. Some of the KPCY tumors had a dense stroma at the center of the tumor as a result of desmoplasia reactions and fibroblasts were found in the core tumor of the KPCY tumors. The infiltration of lymphoid cells was significantly lower within the densest tumor regions compared to the rest of the tumor. The tumors with large amounts of connective tissue (KPCY and CMT167) appeared to have lymphoid cells that were more spread out in the plane than the models with less connective tissue (4T1 and MC38). In general, the tumors all resembled human cancers to some degree albeit highly proliferative and poorly differentiated tumors.

### 3.2 Immune cell populations

**FIGURE 4:**
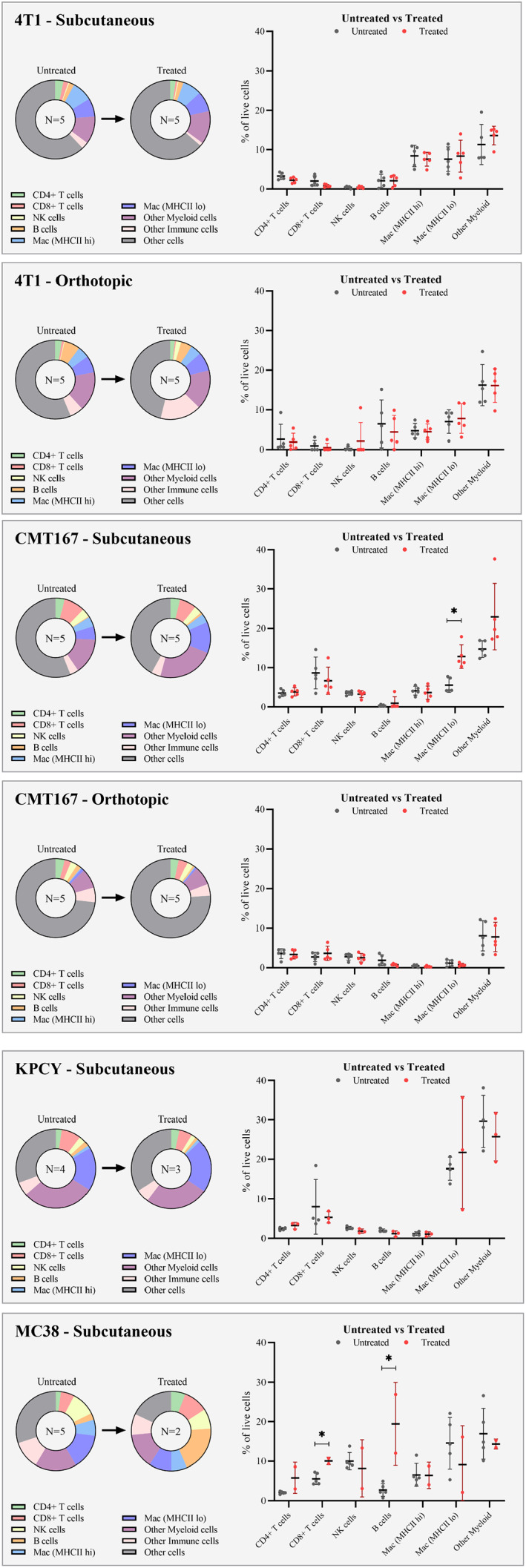
FCM results displaying immune cell populations for the different tumor models, reported as % of live cells. Untreated and treated groups are compared for each model.

The native immune cell population differed between both cell type and implantation site. The distribution of cells is shown in Figure 4, and exact percentages in Table 4. For the subcutaneous models, the highest number of immune cells was observed for MC38 (70%) and KPCY (69%), and the lowest for CMT 167 (44%) and 4T1 (38%).

**TABLE 4:** Cell populations from flow cytometry for untreated tumors of the various models.

Model/Cell population	4T1 OT	4T1 SC	CMT 167 OT	CMT 167 SC	KPCY SC	MC38 SC
CD3+ T cells	3.9 ± 5.3	5.8 ± 1.3	7.3 ± 2.5	13.9 ± 5.1	10.9 ± 6.8	8.4 ± 1.5
CD8+ T cells	0.9 ± 1.4	2.0 ± 1.3	2.7 ± 1.2	8.6 ± 4.1	8.0 ± 6.9	5.6 ± 1.4
CD4+ T cells	2.6 ± 3.8	3.3 ± 0.8	3.6 ± 1.2	3.5 ± 0.9	2.4 ± 0.4	2.1 ± 0.3
NK cells	0.3 ± 0.5	0.4 ± 0.2	2.8 ± 0.8	3.5 ± 0.5	2.7 ± 0.4	10.0 ± 2.2
B cells	6.5 ± 6.0	2.1 ± 1.7	1.9 ± 1.3	0.4 ± 0.2	2.0 ± 0.4	2.8 ± 1.6
MHCII high macrophages	4.7 ± 1.8	8.4 ± 2.7	0.5 ± 0.3	4.1 ± 1.1	1.2 ± 0.5	6.6 ± 2.9
MHCII low macrophages	7.0 ± 3.0	7.6 ± 3.1	1.1 ± 0.8	5.5 ± 1.8	17.6 ± 2.9	14.6 ± 6.5
Other myeloid cells	16.2 ± 5.2	11.3 ± 5.1	8.1 ± 3.8	14.7 ± 2.1	29.6 ± 6.6	17.0 ± 6.4
Other immune cells^a^	5.5 ± 2.7	2.8 ± 2.1	6.0 ± 1.8	3.5 ± 1.3	5.5 ± 1.6	11.5 ± 5.4
Other (non‐immune) cells.	56.3 ± 8.8	62.1 ± 4.9	73.3 ± 10.4	56.0 ± 9.3	30.9 ± 4.2	30.0 ± 9.8

^a^ Includes CD3+CD4−CD8− T cells.

For lymphoid cells (T cells, NK cells and B cells), the 4T1 model had a significantly lower fraction (8.3% of live cells) compared to CMT 167 (17.8%), KPCY (15.6%), and MC38 (21.2%). When inspecting individual cell populations, the 4T1 model was found to have fewer T cells (all CD45+CD3+ cells) (5.8% of live cells) compared to CMT 167 (13.9%) and KPCY (10.9%). The amount of T cells in the MC38 model was not significantly different from the other models (8.4%). However, the MC38 model was found to have more NK cells (10.0% of live cells) than the other models (less than 4% for all the other models). The fraction of T cells being CD8+ was furthermore found to be lower in the 4T1 tumors (34% of T cells) compared to KPCY (73%), MC38 tumors (66%) and CMT 167 (62% of T cells).

The total % of myeloid cells (MHCII high macrophages, MHCII low macrophages, and other myeloid cells) was significantly higher in KPCY and MC38 tumors than in CMT 167 and 4T1 tumors. The % of macrophages was found to be lowest in CMT 167 tumors (9.7%) and was significantly lower than both KPCY (18.9%) and MC38 (21.1%). 4T1 also had a higher mean percentage of macrophages than CMT 167 (16.0%), but the difference was insignificant. MHCII high macrophages were found to account for a substantially lower fraction of all macrophages in the KPCY model (6%) compared to CMT 167 (43%), 4T1 (53%), and MC38 (31%).

The orthotopic CMT 167 had significantly fewer immune cells (26.7% of live cells) compared to the subcutaneous model (44.0%), partly caused by large amounts of normal cells in the samples. Orthotopic samples had significantly lower % of all myeloid cell populations compared to the subcutaneous samples, including all macrophages (1.6% of live in orthotopic vs. 9.7% in subcutaneous), MHCII high macrophages (0.5% vs. 4.1%), MHCII low macrophages (1.1% vs. 5.5%), and other myeloid cells (8.1% vs. 14.7%). In addition, the orthotopic samples displayed significantly lower % of T cells (7.3% vs. 13.9%) and CD8+ T cells (2.7% vs. 8.6%). The % of B cells was, on the other hand, significantly higher in the orthotopic model compared to the subcutaneous model (1.9% vs. 0.4%). NK1.1 cells and CD4+ T cells were the only populations with no difference between the models.

The orthotopic 4T1 model showed similar immune landscapes as the subcutaneous tumors. The only difference was a slightly higher amount of MHCII high macrophages in the subcutaneous tumors (8.4% of live cells) compared to orthotopic tumors (4.7%). Furthermore, the orthotopic tumors had a higher variation in the amount of different immune cells compared to the subcutaneous model.

### 3.3 Effect of PD1 treatment

Effect of PD‐1 treatment is summarized in Table 5 and shown for each cell type in each model in Figure 4. Variable responsiveness toward anti‐PD1 was observed, ranging from the most responsive MC38 colon cancer model to the least responsive 4T1 breast cancer model. There was no effect of anti‐PD1 on the tumor growth of 4T1, CMT 167, or KPCY models, while complete remission occurred in three of five treated animals bearing MC38 tumors.

**TABLE 5:** Summary of the effect of PD‐1 treatment. Time to reach endpoint is displayed for each tumor model, along with number of animals in complete remission per group.

	Time to reach endpoint	Complete remission	Effect on immune landscape
4T1 OT	14 days	0/5	No changes
4T1 SC	10 days	0/5	Reduced density of T cells from FCM
CMT 167 OT	15 days	0/5	No changes in FCM (increased T cells in CLSM)
CMT 167 SC	18 days	0/5	Increased density of myeloid cells from FCM
KPCY SC	15 days	0/5	No changes in FCM (increased T cells in CLSM)
MC38 SC	17 days	3/5	Increased density of T cells and B cells in FCM

There were no significant differences in cell populations between untreated and treated KPCY tumors. For CMT 167, differences in immune cell populations between treated and untreated tumors were only observed for subcutaneous tumors. The CMT 167 subcutaneous tumors that received anti‐PD1 treatment were observed to have an increased amount of MHCII low macrophages (12.8% vs. 5.5%) compared to the untreated tumors. There was also an (insignificant) increase in the mean % of other myeloid cells (23.0% vs. 14.7%). For the orthotopic model, no significant differences were observed in FCM.

For the 4T1 model, differences in immune cell populations between treated and untreated tumors were only observed for subcutaneous tumors, where the % of T cells (CD45+, CD3+) was significantly lower in the treated tumors (5.8% of live cells in untreated vs. 3.3% in treated tumors). The amount of CD8+ T cells was also lower for the treated group (2.0% of live cells in untreated vs. 0.8% in treated tumors). However, this difference was not significant.

For the MC38 tumors, there was a significantly higher fraction of lymphoid cells (total amount of T cells, NK cells, and B cells) in the treated tumors (44.6% of live cells) compared to untreated tumors (21.2%). Significantly higher % were observed explicitly for T cells (16.9% of live cells in treated vs. 8.4% in untreated), CD8+ T cells (10.0% of live cells in treated vs. 5.6% in untreated), and B cells (19.5% of live cells in treated vs. 2.8% in untreated). The average % of CD4+ T cells was also noted to be higher in the treated group, but this was not significant (5.8% of live cells in the treated vs. 2.1% in the untreated). The amount of NK cells was not significantly different either, with a slightly lower mean in the treated group (8.2% of live cells in treated vs. 10.0% in untreated). One of the treated tumors was noted to have a low amount of MHCII macrophages (2.2%) compared to the average of untreated tumors (14.6%). However, this was not observed for the other treated tumor, and the comparison between the treated and untreated tumors revealed no significant difference.

### 3.4 Distribution of T cells

**FIGURE 5:**
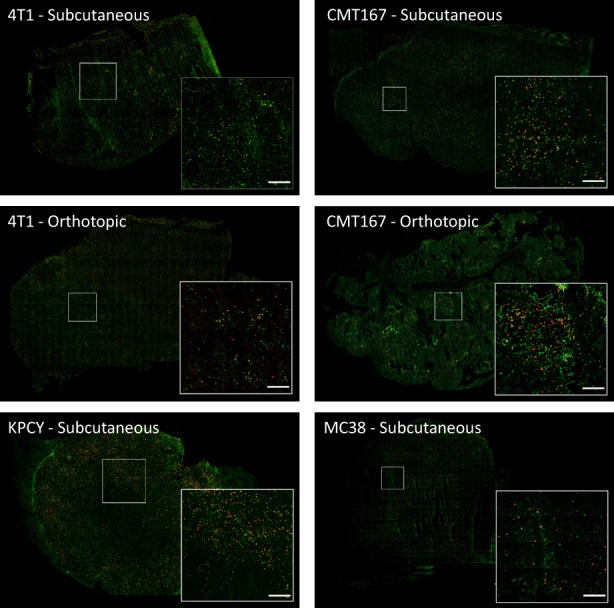
CLSM images showing the distribution of CD3 (green) and CD8 (red) positive T cells in the various tumor models. Scale bars are 200 μm.

**FIGURE 6:**
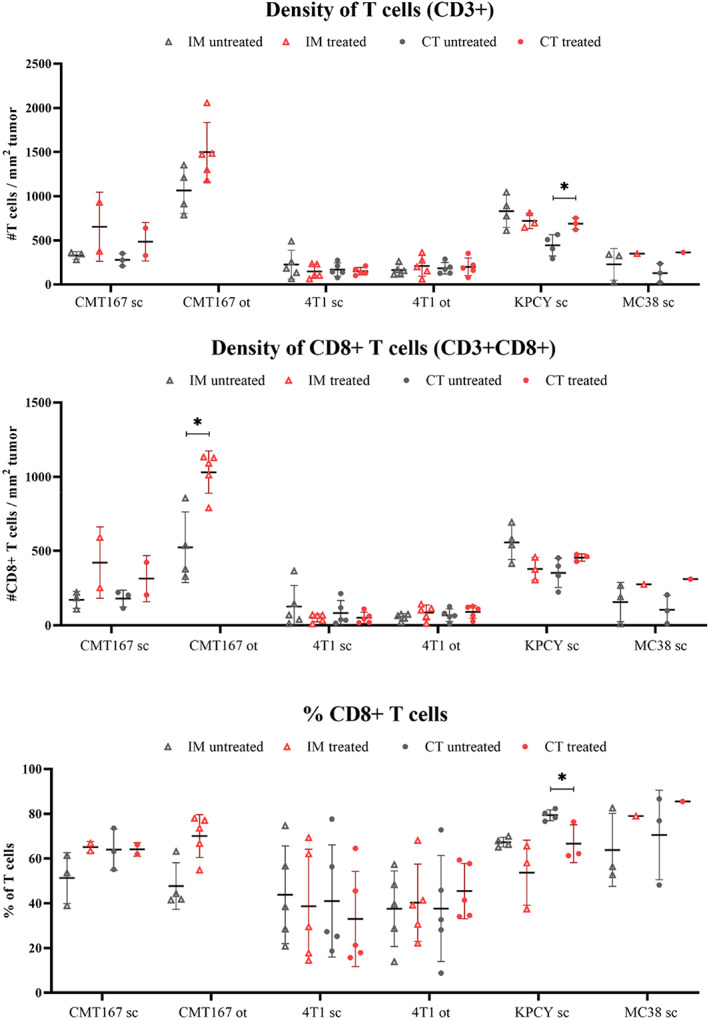
CLSM analysis of the different treated and untreated tumor models displaying the amount of CD8 and CD3 positive cells, in addition to the percentage of CD8 positive cells, in the tumor core (CT) and the invasive margin (IM).

The distribution of T cells was analyzed by fluorescence microscopy of tumor sections. Representative images are shown in Figure 5 and the resulting data from image analysis in Figure 6. When comparing the four different subcutaneous models, the subcutaneous KPCY tumors had the highest density of T cells and CD8+ T cells, significantly higher than in 4T1 and MC38 tumors (CT and IM). The density of T cells and CD8+ T cells in the IM of KPCY tumors was also higher than in the IM of CMT 167 tumors. A general trend was observed that the mean density of T cells was higher in the IM than in the CT, but the difference was only significant for the KPCY tumors.

For CMT 167, the CT and IM regions were evaluated for the subcutaneous tumors. For the orthotopic model, all tumor tissue was evaluated as one region (tumor) due to multiple small tumors in the tissue. The baseline infiltration of T cells was found to be significantly higher in orthotopic tumors (1065 T cells/mm^2^ tumor) than in both CT and IM regions of the subcutaneous tumors (CT: 279 T cells/mm^2^ tumor, IM: 328 T cells/mm^2^ tumor). No significant differences were found between the models regarding the density of CD8+ T cells or the fraction of CD8+ T cells (% of T cells). A significant increase in the density of CD8+ T cells was observed in treated, orthotopic tumors from 524 cells/mm^2^ (untreated) to 1031 cells/mm^2^ CD8+ T cells/mm^2^ tumor (treated). An elevated density of CD8+ T cells was also observed in the subcutaneous model, which was most pronounced in the invasive margin of the tumor (328 CD8+ T cells/mm^2^ in untreated tumors to 654 cells/mm^2^ in treated tumors). However, the elevated infiltration of CD8+ T cells in treated subcutaneous tumors was not significant.

For 4T1 tumors, the CT and IM regions were evaluated separately in both subcutaneous and orthotopic tumors. CLSM data showed no significant difference between subcutaneous and orthotopic 4T1 tumors in terms of the density of T cells, the density of CD8+ T cells, or the fraction of CD8+ T cells. There was no significant difference between CT and IM in either model, and no effect of treatment with PD1.

The untreated KPCY tumors were, as presented earlier, found to have significantly more T cells in the invasive margin compared to the core tumor region. However, this difference in infiltration between the two regions was not observed for the treated tumors. The treated tumors were found to have an increased density of T cells within the CT (688 CD8+ T cells/mm^2^ tumor in treated vs. 443 in untreated). Furthermore, a decrease in the fraction of CD8+ T cells within the IM was found (79.3% of T cells in untreated vs. 66.6% in treated).

Only one MC38 tumor was evaluated with CLSM analysis, precluding any statistical analyses. However, it is noted that the treated, and responding, tumor had a higher density of CD8+ T cells in the CT region than all the untreated tumors (311 CD8+ T cells/mm^2^ tumor in treated vs. 105 in untreated).

## 4 DISCUSSION

The cellular immune landscape of untreated tumors of the four tumor models investigated (CMT 167, 4T1, KPCY, and MC38) demonstrated significant differences. The subcutaneous KPCY and MC38 tumors had the most immune cells, with a significantly higher infiltration of myeloid cells than the subcutaneous 4T1 and CMT 167 tumors. High infiltration of T cells was observed in the subcutaneous KPCY model and the orthotopic CMT 167 model, intermediate in the subcutaneous CMT 167 model, and low in the MC38 and 4T1 models. The amount of connective tissue was furthermore noted to be higher in subcutaneous CMT 167 and KPCY than in 4T1 and MC38.

Anti‐PD1 treatment was found to affect the cellular immune landscape of the tumor models to a varying degree. The MC38 model was most responsive with complete remission in 60% of the treated tumors, and increased infiltration of CD8+ T cells and B cells. This is in accordance with previous studies showing that this model responds to checkpoint inhibition.^8^, ^9^, ^10^, ^11^ The importance of CD8+ T cells in response to PD‐1 treatment was also observed by others,^9^, ^12^ while the role of B cells has been discussed.^13^, ^14^, ^15^, ^16^, ^17^ Previous studies have reported that response toward checkpoint inhibition could be connected to a combination of increased infiltration of CD8+ T cells and a decreased infiltration of myeloid cells, such as macrophages.^9^, ^18^ Subtypes of myeloid cells have been associated with pro‐tumor effects and poor responses.^19^ In line with our findings, Taylor et al. also demonstrated that increased CD8+ T cells and decreased F4/80+ macrophages were linked to PD‐1 responsive tumors in another murine colon cancer model.^18^

Based on confocal images, the CMT 167 model was found to be moderately responsive with increased infiltration of CD8+ T cells especially in the orthotopic model as observed also by others.^20^, ^21^ The same was not observed with FCM, but for this model specifically imaging is probably more accurate as it only evaluates the metastases and not the healthy lung. The subcutaneous model displayed increased infiltration only of myeloid cells, indicating that it could be less responsive than the orthotopic one.^20^, ^21^

Some of the similarities in immune profile of 4T1 and MC38, the least and most responsive tumor in the study shows that therapeutic response cannot be predicted reliably by the presence of certain types of immune cells alone. Accordingly, high baseline infiltration of CD8+ T cells is neither enough nor a prerequisite for a robust response toward anti‐PD1. Robust responses were connected to increased infiltration of CD8+ T cells. However, there is likely a balance between the anti‐tumor effects exerted primarily by cytotoxic CD8+ T cells and model‐specific immunosuppressive mechanisms, such as myeloid cells, regulatory adaptive immune cells, and physical barriers such as excessive connective tissue. KPCY exhibited the highest baseline infiltration of CD8+ T cells but did not respond readily to therapy based on FCM data. An increased amount of T cells was observed in CLSM images. The specific clone of KPCY used in this study was selected as a subcategory of KPCY with high T‐cell infiltration^6^ and responds in the original study to a combination regimen with gemcitabine, nab‐paclitaxel, anti‐CD40, anti‐CTLA‐4 and anti‐PD1.^6^ The high infiltration of T cells differs from other KPC models which do not respond to checkpoint inhibition, with poor infiltration of T cells, high infiltration of macrophages and cancer‐associated fibroblasts.^22^, ^23^, ^24^, ^25^, ^26^

While high T‐cell infiltration is no guarantee of response, a particularly low infiltration of CD8+ T cells combined with elevated levels of immunosuppressive and regulatory cells could indicate poor responsiveness, as seen in the 4T1 model. In 4T1 no direct indications of response were observed, only a small reduction in T cells for the treated subcutaneous model. In line with this, multiple studies have previously reported that anti‐PD1 treatment does not affect tumor progression and survival in 4T1 bearing mice.^27^, ^28^ This could be due to poor expansion of T cells in combination with myeloid suppressor cells.^18^, ^27^

The tumor models presented here can be useful in different situations. MC38 is highly responsive and could be used in combination studies with checkpoint inhibition and other treatments, perhaps with a reduced dose of PD‐1 antibody. KPCY and CMT 167 react, but do not readily respond to the therapy and could hence also be good alternatives for hypothesis testing of combinations with checkpoint treatment. For KPCY, the combination used by Li et al.^6^ could work as a positive control. These two models possibly also represent a more realistic physiology with extensive connective tissue around the cancer cells forming both an extracellular matrix and acting as a barrier to drug delivery. Hence, these models could be useful in the study of novel drug delivery approaches. 4T1 appears to be an immunologically quite “cold” tumor^29^ and could be suited for studies combining immunotherapy with radiation or chemotherapy.

While this study provides an overview of the immune landscape in the listed tumor models, it also has limitations that should be considered when using this information to plan future studies. First, the number of animals is kept to a minimum in each group, although larger groups would give more robust statistical analysis. The KPCY tumors grew in only seven of 10 animals and in MC38 three of five mice were in complete remission. The latter also skews the results toward the less responsive tumors. Another limitation is the number of antibodies used in the flow cytometry analysis. The current study separates major cell types, however especially the myeloid populations are difficult to separate well based on single markers. Other antibody panels could be designed to provide more certain classification of selected cell types.

## 5 CONCLUSION

The possibility of modulating an anti‐cancer immune response using checkpoint inhibitors represents a promising opportunity, and murine syngeneic models are an important tool for evaluation of such therapies. The six tumor models studied here displayed significantly different immune landscapes, and variable responsiveness toward anti‐PD1, ranging from the most responsive MC38 colon cancer model to the least responsive 4T1 breast cancer model. Robust responses were connected to increased infiltration of CD8+ T cells. However, a high infiltration of CD8+ T cells in untreated tumors was not directly predictive of a favorable response. There is likely a balance between the anti‐tumor effects exerted primarily by cytotoxic CD8+ T cells and model‐specific immunosuppressive mechanisms, such as myeloid cells, regulatory adaptive immune cells, and physical barriers such as excessive connective tissue. The study provides a foundation for appropriate selection of relevant tumor models to be used for combination treatment studies, and for further elucidation of variable responses toward immunotherapies.

## AUTHOR CONTRIBUTIONS

**Sofie Snipstad:** Conceptualization (equal); methodology (equal); supervision (equal); writing – original draft (equal); writing – review and editing (equal). **Frida Bremnes:** Data curation (equal); formal analysis (equal); methodology (equal); writing – review and editing (equal). **Mats Dehli Haugum:** Data curation (equal); formal analysis (equal); writing – review and editing (equal). **Einar Sulheim:** Conceptualization (equal); methodology (equal); project administration (equal); supervision (equal); writing – original draft (equal); writing – review and editing (equal).

## Data Availability

Raw data will be made available upon request.
